# Synthesis and Properties of Cyclopentyl Cardo-Type Polyimides Based on Dicyclopentadiene

**DOI:** 10.3390/polym11122029

**Published:** 2019-12-06

**Authors:** Shih-Chieh Yeh, Jen-Yu Lee, Chung-Ta Hsieh, Ya-Chin Huang, Kuan-Syun Wang, Chien-Hsin Wu, Chien-Chieh Hu, Shu-Chen Chiang, Ru-Jong Jeng

**Affiliations:** 1 Institute of Polymer Science and Engineering, National Taiwan University, Taipei 106, Taiwan; in93imid@gmail.com (S.-C.Y.); so850306w@gmail.com (J.-Y.L.); dd7954613@gmail.com (C.-T.H.); pley810030@gmail.com (Y.-C.H.); wting830606@gmail.com (K.-S.W.); 2 Advanced Research Center for Green Materials Science and Technology, National Taiwan University, Taipei 106, Taiwan; 3 Graduate Institute of Applied Science and Technology, National Taiwan University of Science and Technology, Taipei 106, Taiwan; 4 Chung Shan Institute of Science and Technology, Taoyuan 325, Taiwan; usb14@hotmail.com

**Keywords:** C5 byproduct, dicyclopentadiene (DCPD), cardo polyimide, gas separation

## Abstract

A crucial polymer intermediate, 4-[1-(4-hydroxyphenyl)cyclopentyl]-phenol (bisphenol CP), was developed from dicyclopentadiene (DCPD), a key byproduct of the C5 fraction in petrochemicals. On the basis of bisphenol CP, a diamine, 4,4′-((cyclopentane-1,1-diylbis(4,1-phenylene))bis(oxy))-dianiline (cyclopentyl diamine; CPDA) was subsequently obtained through a nucleophilic substitution of bisphenol CP, followed by the hydrogenation process. By using the CPDA diamine, a series of polyimides with cyclopentyl (cardo) units on the backbone were prepared along with a reference polyimide (API-6F) based on 4,4′-(4,4′-(propane-2,2-diyl)bis(4,1-phenylene))bis(oxy)dianiline (BPAA), and 4,4′-(hexafluoroisopropylidene)-diphthalic anhydride (6FDA) for the exploration of structure-properties relationship. Thanks to the presence of cyclopentyl units, this type of cardo polyimides exhibited comparable tensile properties, especially a large elongation (25.4%). It is also worth noting that CPI-6F exhibited better solubility in organic solvents, such as NMP, DMAc, THF, and chloroform, than the other PIs. Gas separation properties were also evaluated for these cardo-type polyimides.

## 1. Introduction

**Figure 1:**
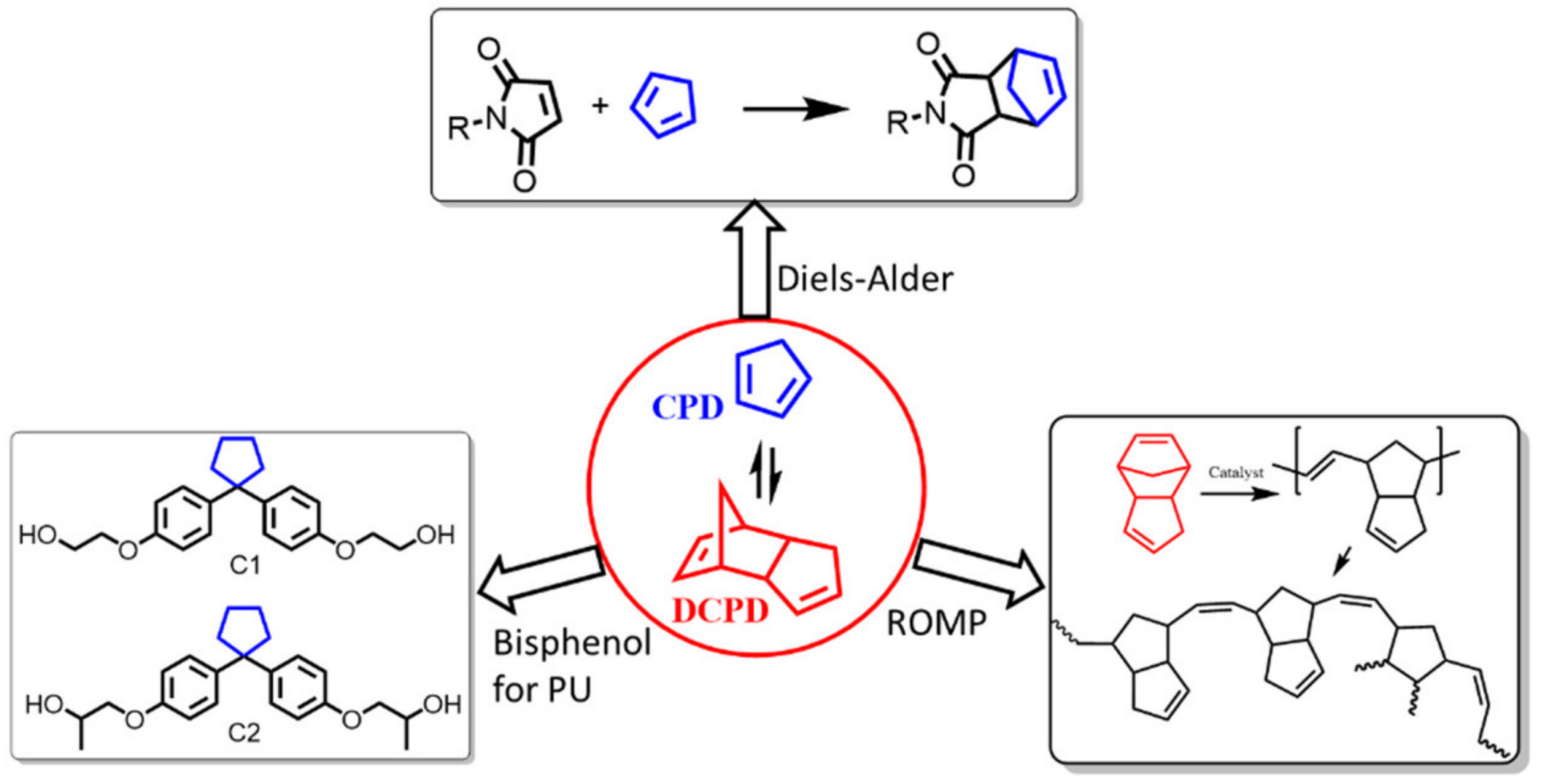
Development of DCPD as a starting material for polymers.

This work is set forth under the premise of high-value petrochemicals and the utilization of wastes from the industry chain. Dicyclopentadiene (DCPD) is one of the C5 byproducts generated in the steam-cracking process of naphtha [1], which is an abundant and inexpensive raw material. It is formed by the Diels–Alder reaction from two cyclopentadiene (CPD) molecules and exists in two stereo-isomers: endo-DCPD and exo-DCPD. The more thermodynamically stable endo-DCPD is usually used as commercial product for a wide range of resins, i.e., aromatic hydrocarbons, unsaturated polyesters, phenolics, and epoxies. Typically, DCPDs can be polymerized with unsaturated hydrocarbons through ring-opening metathesis polymerization (ROMP) method by using proper catalysts [2,3,4,5,6] or Diels–Alder reaction [7,8,9,10]. Nevertheless, the feasibility and diversity of converting DCPD into useful intermediates still has been sought after actively to extend their utilities into polymers with special performances (Figure 1) [1,3,4,5,7]. For example, the use of DCPD provided a feasible pathway in the production of 1,1-cyclopentylenylbisphenol (bisphenol CP), a versatile polymer intermediate [1]. The incorporation of five-membered cardo-type groups onto the polyurethane (PU) backbone through alkoxyl diols of bisphenol CP also significantly enhanced the phase mixing of PU hard and soft segments, leading to a feature of shape memory materials. On the other hand, the incorporation of some cardo (Latin word for “loop”) structures, such as diamantane [11], methanohexahydroindane [12], cyclododecylidene [13], norbornane [14], or cyclopentyl [15] groups, into the polyimide (PI) backbone has been confirmed to be capable of enhancing solubility, mechanical and thermal properties. It is also suggested that the intermolecular chain distance and chain rigidity of the polymer were increased. This is because those bulky alicyclic structures restrict the dense packing and free rotation of the polymer chains. In addition, PIs with good mechanical properties, high thermal stability, and excellent film integrity (defect free) are of great potential for commercial products such as high temperature adhesive tapes, alignment layers for liquid crystal display (LCD) [16,17,18,19,20], etc. PIs also played an important role in the applications of polyelectrolytes (for organic memory devices) [21,22], gate insulators (for organic thin film transistors; OTFTs) [22,23,24,25,26], membranes (for gas separation) [22,27], etc.

**Scheme 1:**
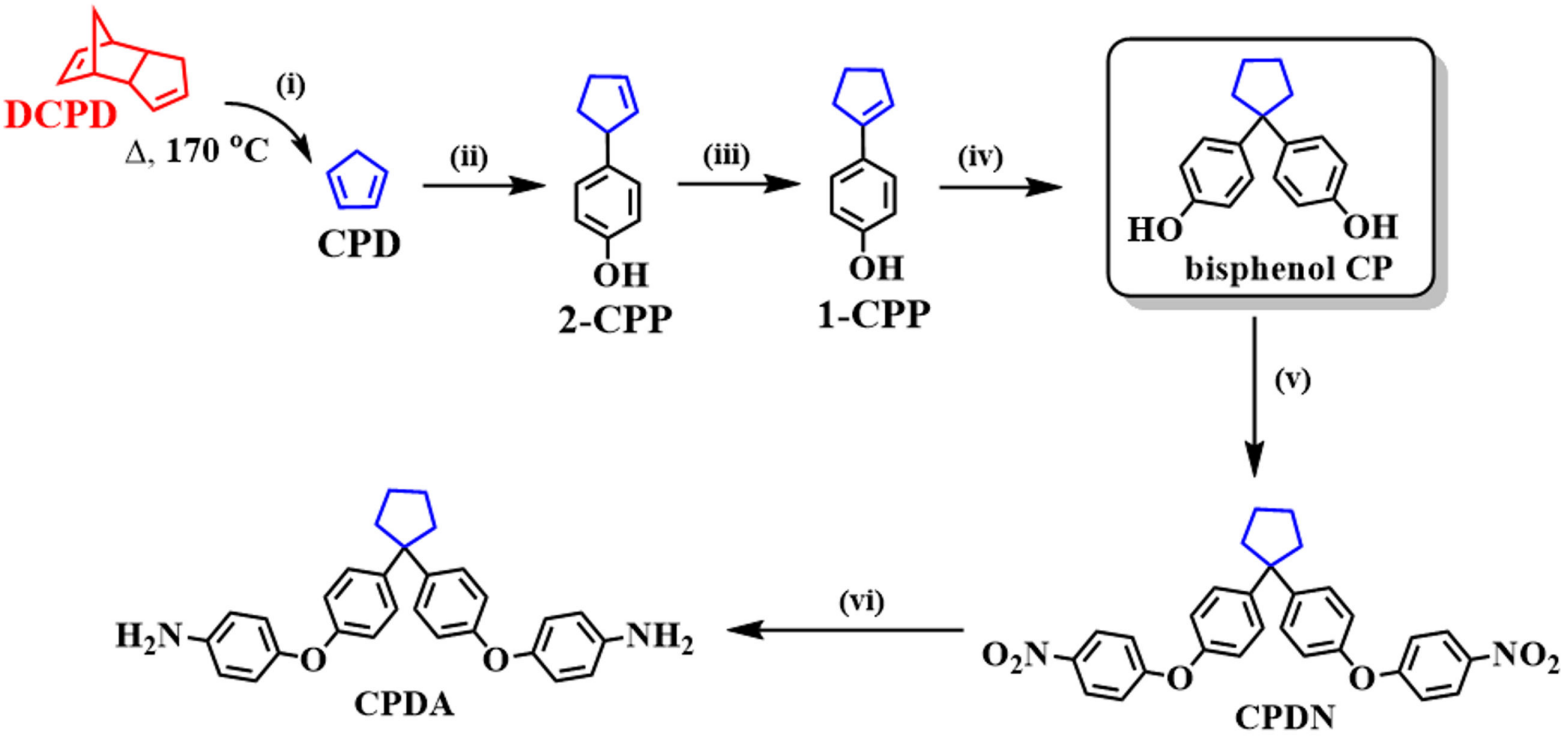
The synthetic route of cardo-type diamine (CPDA): (i) distillation at 170 °C. (ii) phenol, H_3_PO_4_ in toluene at room temperature for 12 h. (iii) PdCl_2_(PhCN)_2_ in toluene for 2 h reflux. (iv) phenol, HCl at 80 °C for 4 h. (v) 4-fluoronitrobenzene in DMAc at 60 °C for 10 h. (vi) hydrazine monohydrate, palladium on carbon 10% in ethanol for 2 h reflux.

**Scheme 2:**
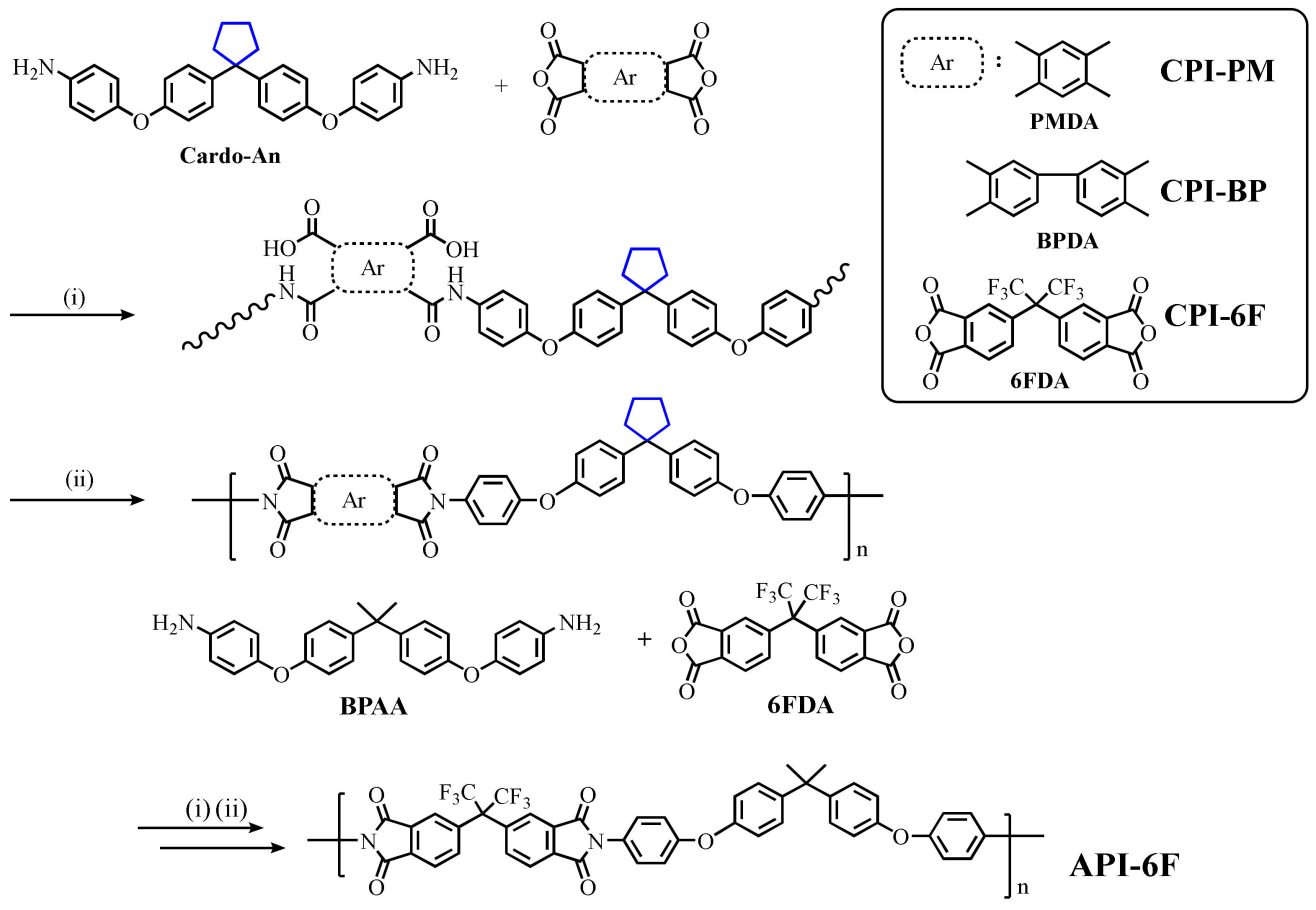
Synthesis of cardo type polyimides: (i) diamine and dianhydride in DMAc at room temperature for 4 h. (ii) PAA solution in petri dish at 60 °C for 5 h, 100 °C for 2 h, 200 °C for 2 h, 250 °C for 20 min.

With the intention of using the recycling C5 byproducts for the synthesis of high-performance PIs, we would like to take advantage of the above-mentioned versatile polymer intermediate, bisphenol CP (Scheme 1) [1]. In this work, a diamine, 4,4′-((cyclopentane-1,1-diylbis(4,1-phenylene))bis(oxy))-dianiline (cyclopentyl diamine; CPDA) was obtained through a nucleophilic substitution of bisphenol CP with p-fluoronitrobenzene, followed by the hydrogenation of dinitro compound, 4,4′-(cyclopentane-1,1-diyl)bis((4-nitrophenoxy)benzene (cyclophentyl dinitro; CPDN). These cardo-type PIs, were then prepared by the condensation of CPDA with pyromellitic dianhydride (PMDA), 3,3′,4,4′-biphenyltetracarboxylic dianhydride (BPDA), and 4,4′-(hexafluoroisopropylidene)-diphthalic anhydride (6FDA), respectively, to form CPI-PM, CPI-BP, and CPI-6F (Scheme 2). For the sake of the comparison, 4,4′-(4,4′-(propane-2,2-diyl)bis(4,1-phenylene))bis(oxy)dianiline (BPAA) was reacted with 6FDA to form a PI, i.e., API-6F. It is important to note that the presence of cyclopentyl (cardo) structures on the main chains of polyimides would exhibit specific free volumes due to the hindrance of dense interchain packing and free rotation of the polymer chains [11,28]. This might be fit for gas membrane separation [29,30].

## 2. Experimental

### 2.1. Materials

Dicyclopentadiene (DCPD), phenol, phosphoric acid (H_3_PO_4_), sodium carbonate (Na_2_CO_3_), bis(benzonitrile)palladium (II) chloride (PdCl_2_(PhCN)_2_), hydrochloric acid (1 M), hexane, N,N-dimethylacetamide (DMAc), toluene, ethanol, potassium carbonate (K_2_CO_3_), NaOH_(aq)_ (1 M), palladium on carbon (Pd/C), 4-fluoronitrobenzene, hydrazine, Pyromellitic dianhydride (PMDA), 3,3′,4,4′-Biphenyltetracarboxylic dianhydride (BPDA), 4,4′-(hexafluoroisopropylidene) diphthalic anhydride (6FDA), 2,2-Bis(4-hydroxyphenyl)propane (bisphenol A; BPA), dimethyl-sulfoxide-d_6_ (DMSO-d_6_), and CDCl_3_ were purchased from Sigma-Aldrich (Darmstadt, Germany) or Acros (Geel, Belgium).

### 2.2. Characterization Methods

Nuclear magnetic resonance (NMR) spectra were detected by a Varian Gemini NMR 400 (Palo Alto, CA, USA) with DMSO-d_6_ or CDCl_3_ as the solvent. IR spectra were recorded on a Jasco 4100 (Baltimore, WA, USA) attenuated total reflection Fourier transform infrared (ATR-FTIR) spectrophotometer in the range of 4000–500 cm^−1^. Wide-angle X-ray diffraction (WAXD) measurement was performed by PANalytical-X’Pert PRO MPD (Malvern Panalytical, EA Almelo, The Netherland) which uses CuKα radiation at a wavelength of 1.54187 Å, operating in a 2θ range of 5°–60°.Thermogravimetric analysis (TGA) at 5% weight loss (*T*_d5_) were conducted with a TA Instruments Q50 (TA Instruments, DE, USA) in nitrogen at a heating rate of 10 °C/min. Glass transition temperature (*T*_g_) was performed by Seiko S II Model SSC5200 (Seiko Instruments Inc., Chiba-shi, Japan) in nitrogen at a heating rate of 10 °C/min. Tensile measurements were performed using a compact table-top universal/tensile tester (Model: MTS Landmark 370.02 Test System, MTS Systems Corporation, Eden Prairie, MN, USA) with a cross-head speed of 100 mm/min. The tensile bars were prepared by following the ASTM D638 standard. SEM cross-section images were obtained on a JEOL field emission scanning electron microscope (FE-SEM) (Model: JSM-6700F, JOEL Ltd., Tokyo, Japan) under a 15 KV accelerating electron voltage, 50 K magnitude.

The gas permeability of the PI membranes was measured with a Yanaco GTR-11MH gas permeation analyzer (Yanaco, Kyoto, Japan). The measurements were carried out under a feed gas pressure of 1 atm at 35 °C. The experimental data was continuous recorded for 10 min after the system reaching steady state. The gas permeability (P) in Barrer (1 Barrer = 1 × 10^−10^ cm _(STP)_^3^ cm/(cm^2^ s cm Hg)) was calculated from the pressure difference between feed and permeate side, under the following specifications: the effective area of the membrane (2.5 cm diameter), and the membrane thickness (50–100 μm). The selectivity (α) is obtained from the relation between the permeation coefficients of pure i and j gases as: α_i/j_ = P_i_/P_j_. The fractional free volume (FFV) of each polymer was estimated from Equation (1) [31,32,33]:

(1) $$\mathrm{FFV}=\;\frac{V-V_{0}}{V}$$

where *V*_0_ is the occupied volume of the polymer and *V* is the specific volume of the PI (i.e., the inverse of the PI density). The density of a PI was determined experimentally using an analytical balance (AX205, Mettler Toledo, Midland, Ontario, Canada) coupled with a density kit. The *V*_0_ was calculated by Bondi’s method, as shown in Equation (2):

(2) $$V_{0}=\;1.3V_{VDW}$$

where *V_VDW_* is the summary of van der Waals volume of each structural group in the PI [34].

### 2.3. Synthesis

#### 2.3.1. Cyclopentadiene (CPD) [1,35,36,37]

Cyclopentadiene was obtained from pyrolysis of DCPD at 170 °C by distillation. Then the liquid product was kept at 0 °C and used without further purification.

#### 2.3.2. Cyclopent-2-enyl phenol (2-CPP) [1,35,36,37]

To a mixture of phenol (94 g, 1 mol), CPD (33 g, 0.5 mol), and 100 mL toluene in the 500 mL two-neck flask, 85% phosphoric acid (115.4 g, 1 mol) was added. The reaction mixture was kept at room temperature and stirred under nitrogen atmosphere for 2 h. Subsequently, the mixture was neutralized with 160 g sodium carbonate (Na_2_CO_3_) and filtrated to collect the crude. After the removal of toluene and phenol, the crude was recrystallized from hexane to afford 2-CPP with 65% (46 g) yield. ^1^H NMR (DMSO-d_6_, 400 MHz): δ 8.27 (d, 4 H, Ar–H), 7.48 (d, 4 H, Ar–H), 7.14 (m, 8 H, Ar–H), 2.36 (s, 4 H, –CH_2_–), 1.69 (s, 4 H, –CH_2_–). ^13^C NMR (DMSO-d_6_, 100 MHz): δ 155.9, 136.7, 135.2, 131.1, 128.1, 115.5, 50.1, 33.9, 32.4.

#### 2.3.3. Cyclopentenyl phenol (1-CPP) [1,35,36,37]

To a mixture of 2-CPP (3 g, 0.01 mol) and 30 mL toluene in the 250 mL two-neck flask, PdCl_2_(PhCN)_2_ (0.15 g) was added. The reaction mixture was kept refluxing under nitrogen atmosphere for 2h. The filtration process was proceeded at reaction temperature to remove the catalyst by Celite, and toluene was subsequently removed by rotation evaporator. The light green product was recrystallized from toluene to afford 1-CPP with 93% (2.79 g) yield. ^1^H NMR (DMSO-d_6_, 400 MHz): 9.43(s, 1 H, –OH), 7.30(d, 2 H, Ar–H), 6.74(d, 2 H, Ar–H), 6.06(s, 1 H, –CH–), 2.62(s, 2 H, –CH_2_–), 2.48(s, 2 H, –CH_2_–), 1.96(s, 2 H, –CH_2_–). ^13^C NMR (DMSO-d_6_, 100 MHz): δ 156.9, 142.0, 127.7, 115.4, 33.2, 33.1, 23.2.

#### 2.3.4. *4,4′*-(Cyclopentane-1,1-diyl)diphenol (bisphenol CP) [1,35,36,37]

To a mixture of 1-CPP (0.5 g, 0.018 mol) and phenol (4 g, 0.042 mol) in the 100 mL two-neck flask, 1 M hydrochloric acid (1 mL) was added. The reaction mixture was stirred at 80 °C for 4h. Subsequently, 1 M NaOH_(aq)_ (50 mL) was added to the mixture dropwise, then participate was washed with large quantities of water. The solid was collected and dried in vacuum oven. After the recrystallization from toluene, the pink powder was afforded with 50% (0.8 g) yield. ^1^H NMR (DMSO-d_6_, 400 MHz): 9.12(s, 2 H, –OH), 7.08(d, 4 H, Ar–H), 6.65(d, 4 H, Ar–H), 2.18(s, 4 H, –CH2–), 1.60(s, 4 H, –CH_2_–). ^13^C NMR (DMSO-d_6_, 100 MHz): δ 155.1, 139.6, 127.7, 127.7, 115.0, 54.3, 38.5, 22.7.

#### 2.3.5. General Synthetic Route of Dinitro Compound: 4,4′-(cyclopentane-1,1-diyl)bis((4-nitrophenoxy)benzene) (CPDN) [38,39]

To a mixture of bisphenol CP (0.5 g, 1.9 mmol), and 1-fluoro-4-nitrobenzene (0.61 g, 4.3 mmol) in the 50 mL two-neck flask, 0.8 g K_2_CO_3_ and 3 mL DMAc were added. The reaction mixture was kept at 60 °C for 10 h. The mixture was added dropwise into ethanol to collect the precipitate. After drying, the brown solid was recrystallized from ethanol to afford CPDN with 85% (0.73 g) yield. ^1^H NMR (DMSO-d_6_, 400 MHz): 8.27(d, 4 H, Ar–H), 7.48(d, 4 H, Ar–H), 7.14(m, 8 H, Ar–H), 2.36(s, 4 H, –CH_2_–), 1.69(s, 4 H, –CH_2_–). ^13^C NMR (DMSO-d_6_, 100 MHz): δ 163.2, 152.6, 145.7, 142.6, 129.1, 126.6, 120.5, 117.8, 55.9, 38.6, 22.7.

#### 2.3.6. General Synthetic Route of Diamine Compound: 4,4′-((cyclopentane-1,1-diylbis(4,1-phenylene))bis(oxy))dianiline (CPDA)

To a mixture of CPDN (0.5 g, 1 mmol), 10% Pd/C (0.04 g), and 30 mL ethanol in the 100 mL two-neck flask, hydrazine monohydrate (5 mL) was added. The reaction mixture was stirred at 65 °C under nitrogen atmosphere for 2 h. Subsequently, the catalyst was removed by Celite, and the residual ethanol was removed under reduce pressure. The solid product was recrystallized from ethanol to afford CPDA with 80% (0.35 g) yield. ^1^H NMR (DMSO-d_6_, 400 MHz): 7.23(d, 4 H, Ar–H), 6.75(m, 8 H, Ar–H), 6.59(d, 4 H, Ar–H), 4.98(s, 4 H, –NH_2_), 2.24(s, 4 H, –CH_2_–), 1.62(s, 4 H, –CH_2_–). ^13^C NMR (CDCl_3_, 100 MHz): δ 157.0, 145.9, 145.7, 142.2, 128.1, 121.3, 116.2, 115.2, 54.5, 38.4, 22.7.

#### 2.3.7. *4,4′*-((Propane-2,2-diylbis(4,1-phenylene))bis(oxy))dianiline (BPAA) [40]

^1^H NMR (DMSO-d_6_, 400 MHz): 7.11(d, 4 H, Ar–H), 6.73(d, 8 H, Ar–H), 6.58(d, 4 H, Ar–H), 4.95(s, 4 H, –NH_2_), 1.57(s, 6 H, –CH_3_). ^13^C NMR (CDCl_3_, 100 MHz): δ 157.1, 146.0, 145.7, 144.1, 128.0, 121.2, 116.2, 115.2, 41.7, 31.0.

#### 2.3.8. General Procedure for Synthesizing Polyimide

The PIs were obtained from the process as shown in the following. For CPI-6F, CPDA (0.437 g, 1 mmol) was completely dissolved in 3 mL dried DMAc and the equimolar 6FDA (0.444 g, 1 mmol) was added in one portion. The mixture was stirred at room temperature for 2 h to form a viscous poly(amic acid) (PAA) solution and then poured onto a petri dish. After the removal of solvent in an oven at 60 °C for 5 h, the PAA film was further thermally imidized by sequential heating treatments at 100 °C for 2 h, 200 °C for 2 h, 300 °C for 20 min. A flexible polyimide film was stripped off from the glass surface after cooling to room temperature.

CPI-PM: FTIR (film, ν, cm^−1^): 1777 (asym str of imide C=O), 1731 (sym str of imide C=O), 1378 (str of imide C–N).

CPI-BP: FTIR (film, ν, cm^−1^): 1775 (asym str of imide C=O), 1723 (sym str of imide C=O), 1372 (str of imide C–N).

CPI-6F: FTIR (film, ν, cm^−1^): 1785 (asym str of imide C=O), 1719 (sym str of imide C=O), 1375 (str of imide C–N).

API-6F: FTIR (film, ν, cm^−1^): 1785 (asym str of imide C=O), 1712 (sym str of imide C=O), 1373 (str of imide C–N).

## 3. Results and Discussion

### 3.1. Synthesis and Characterization

In this study, the diamine monomer (CPDA) for cardo polyimide was prepared by using the byproducts in the steam cracking process of C5 fraction as shown in the Scheme 1. The synthetic procedure was based on the facile route for the preparation of the 1,1-cyclopentylenyl bisphenol (bisphenol CP), a versatile intermediate with five-membered cardo group, in our previous endeavor [1]. By the thermal cracking procedure, cyclopentadiene (CPD) was obtained from dicyclopentadiene (DCPD). Subsequently, phenol was directly alkylated with CPD in the presence of phosphoric acid at room temperature. The alkylated CPD (4-(cyclopenten-2-yl)-phenol, 2-CPP) was subjected to isomerization (1-CPP), and alkylation reactions to afford the intermediate of bisphenol CP.

CPDA was then prepared in a two-step sequence by using bisphenol CP. In the first step, the aromatic nucleophilic substitution reaction of bisphenol CP and p-fluoronitrobenzene in the presence of K_2_CO_3_ as catalyst to obtain the dinitro-moiety containing compound (CPDN). CPDN was further reduced by using hydrazine monohydrate in the presence of palladium on carbon (Pd/C) to afford the diamine compound (CPDA). ^1^H, ^13^C NMR spectra for diamine compounds are shown in Figures S1–S4 from Supplementary Material. The chemical structure of CPDN was confirmed by the appearance of 7.14–8.27 ppm (aryl signals) and 2.36 and 1.28 ppm (feature cyclopentyl protons) in ^1^H-NMR spectrum. All the corresponding ^1^H-NMR signals were shifted upfield after the reduction of CPDN to CPDA. Moreover, CPDA was also analyzed by ^13^C-NMR, and the result is exactly same as that reported by You et al. [15]. In You’s work, bisphenol CP was derived from a totally different synthetic route starting from cyclopentanone.

**Figure 2:**
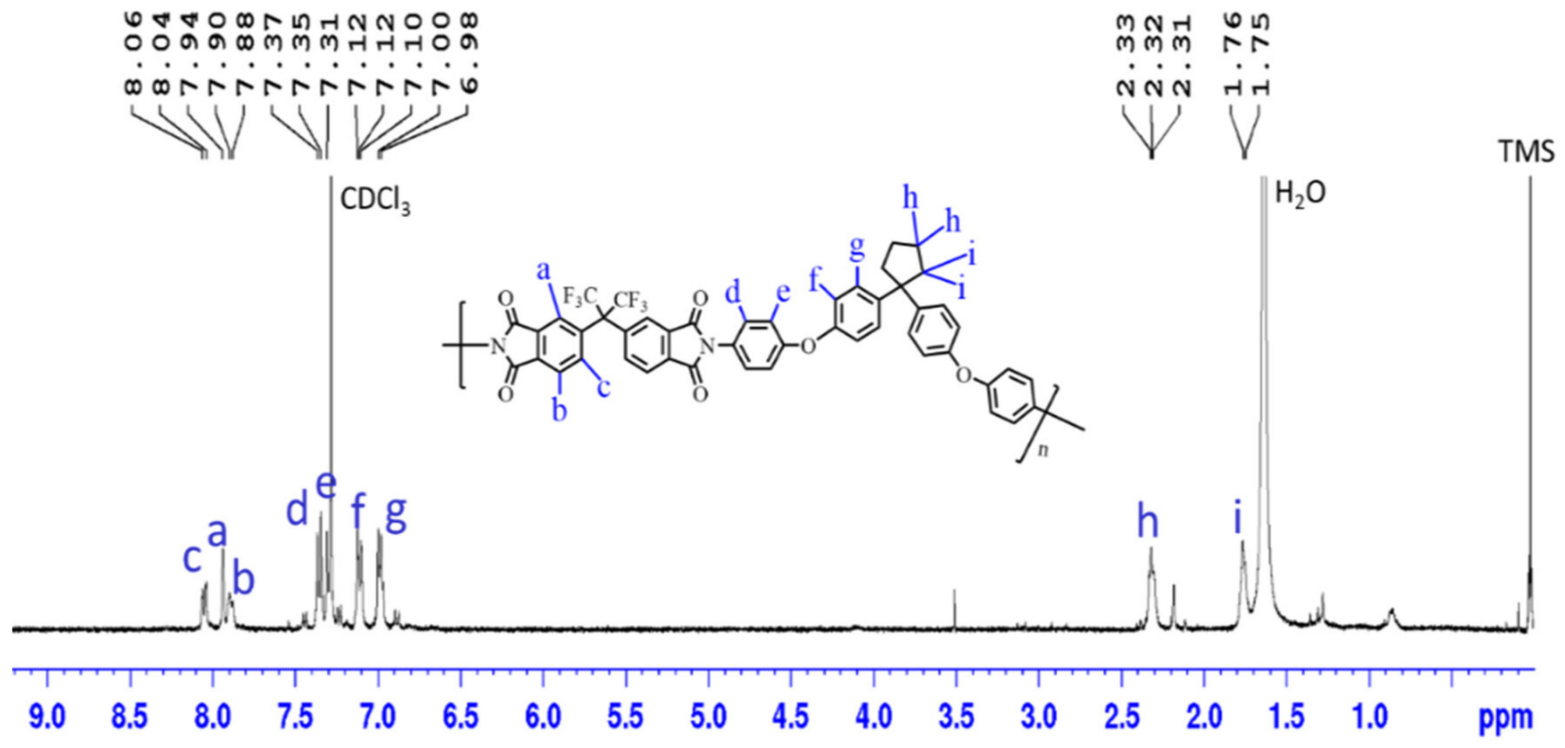
^1^H NMR spectrum of CPI-6F in CDCl_3_ (trimethylsilane (TMS) as reference peak).

**Figure 3:**
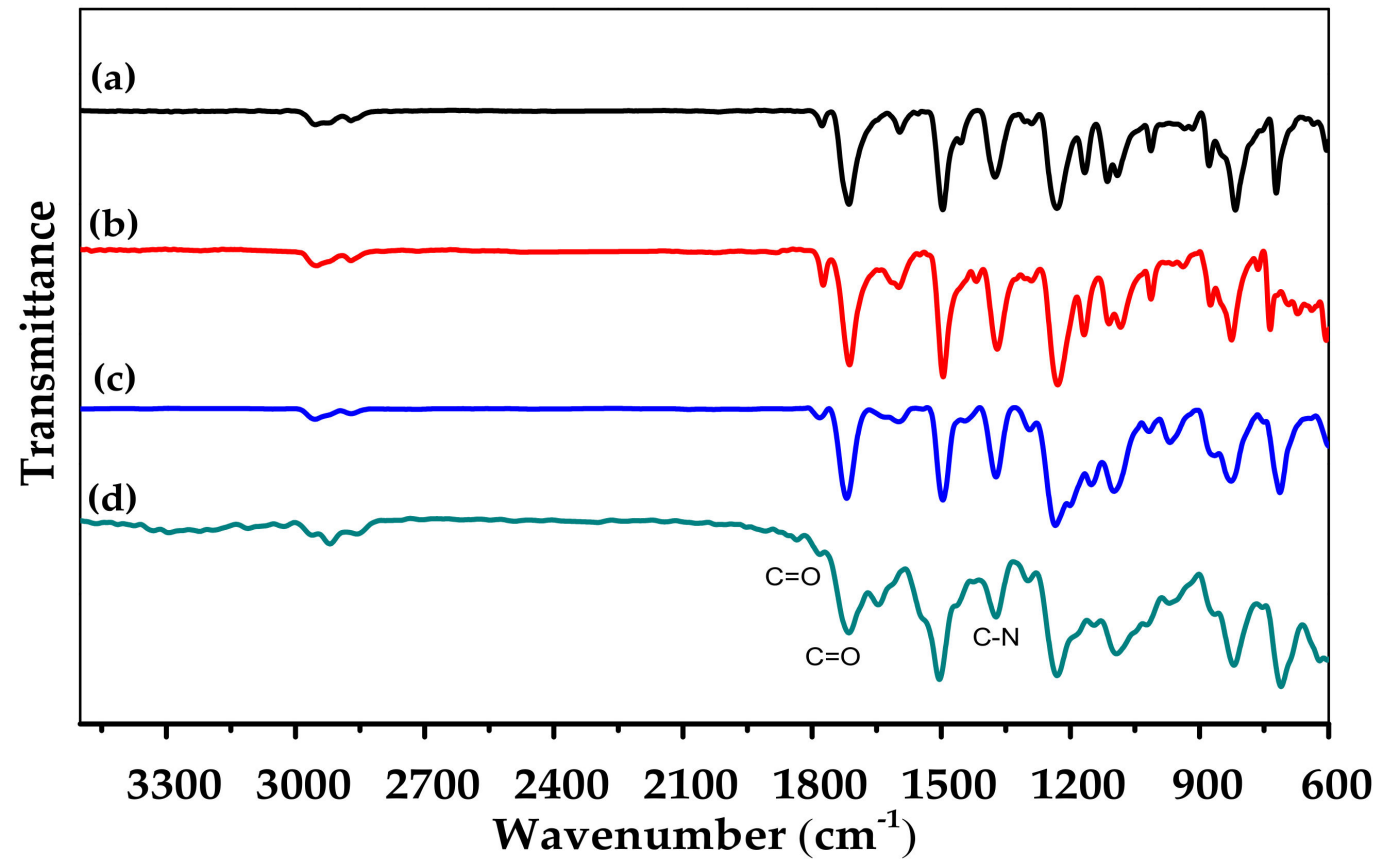
ATR-FTIR spectra of the PI membranes: (**a**) CPI-PM, (**b**) CPI-BP, (**c**) CPI-6F, and (**d**) API-6F.

The polyimides were prepared by the thermal imidization of poly(amic acids) (PAAs) as shown in Scheme 2. PAAs were prepared by the reaction of CPDA diamine with PMDA, BPDA, and 6FDA dianhydrides, respectively, in DMAc solution. For comparison, API-6F with isopropylidene dimethyl between two phenylenes were prepared by BPAA diamine and 6FDA dianhydride under the same condition. The inherent viscosities of these PAAs were measured to be 0.99, 1.27, 0.85, and 0.90 dL/g for CPI-PM, CPI-BP, CPI-6F, and API-6F, respectively. Subsequently, these PAA solutions were respectively casted on the petri dishes to remove solvent under 60 °C for 5 h. The casted PAA films were then thermally imidized at 100 °C for 2 h, 200 °C for 2 h, and 250 °C for 20 min to obtain freestanding PI films. Solubility of these PIs was investigated in different solvents such as NMP, DMF, DMAc, THF, and chloroform (Table 1). It is important to note that the rigidity of these anhydride moieties on PI backbones is in the order of PMDA > BPDA > 6FDA. Consequently, CPI-6F exhibited better solubility in organic solvents than did the other PIs. Moreover, CPI-PM and CPI-BP with relatively rigid units possessed limited solubility in most organic solvents. Figure 2 is ^1^H NMR spectra of CPI-6F, in which all the signals have been assigned to the protons of the repeating unit. The aromatic protons of 6FDA dianhydride moiety were resonated in 8.05, 7.94 and 7.89 ppm for the positions of c, a, and b, respectively. The protons of CPDA diamine moiety for aromatic protons were resonated in 7.36, 7.30, 7.11, and 6.99 ppm for the positions of d, e, f, and g, respectively. The five-membered ring protons of diamine moiety were 2.31 and 1.76 ppm for the positions of h and i, respectively. In addition, PIs were further characterized by FTIR (Figure 3). The absence of 3252 cm^−1^ of acid hydroxyl group, 1712 cm^−1^ of acid carbonyl group, and 1620 cm^−1^ of amide carbonyl group, along with the presence of 1370–1375 cm^−1^ (C–N stretching of imide group), 1715 cm^−1^ (asymmetrical stretching of imide group), and 1778–1785 cm^−1^ (symmetrical stretching of imide group) indicate the imidization of PAAs.

**Figure 4:**
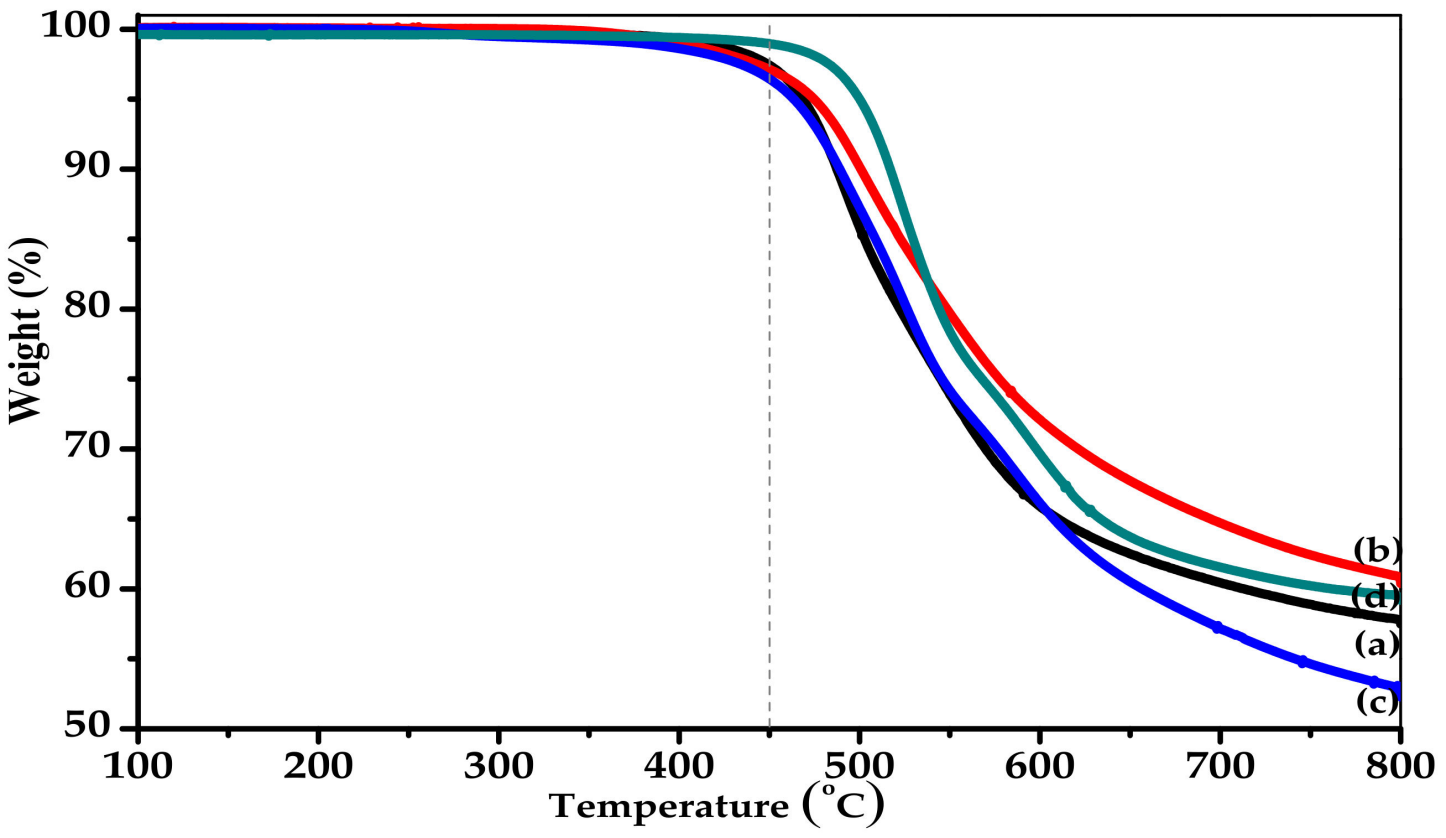
Thermogravimetric analysis of PIs: (**a**) CPI-PM, (**b**) CPI-BP, (**c**) CPI-6F, and (**d**) API-6F.

**Figure 5:**
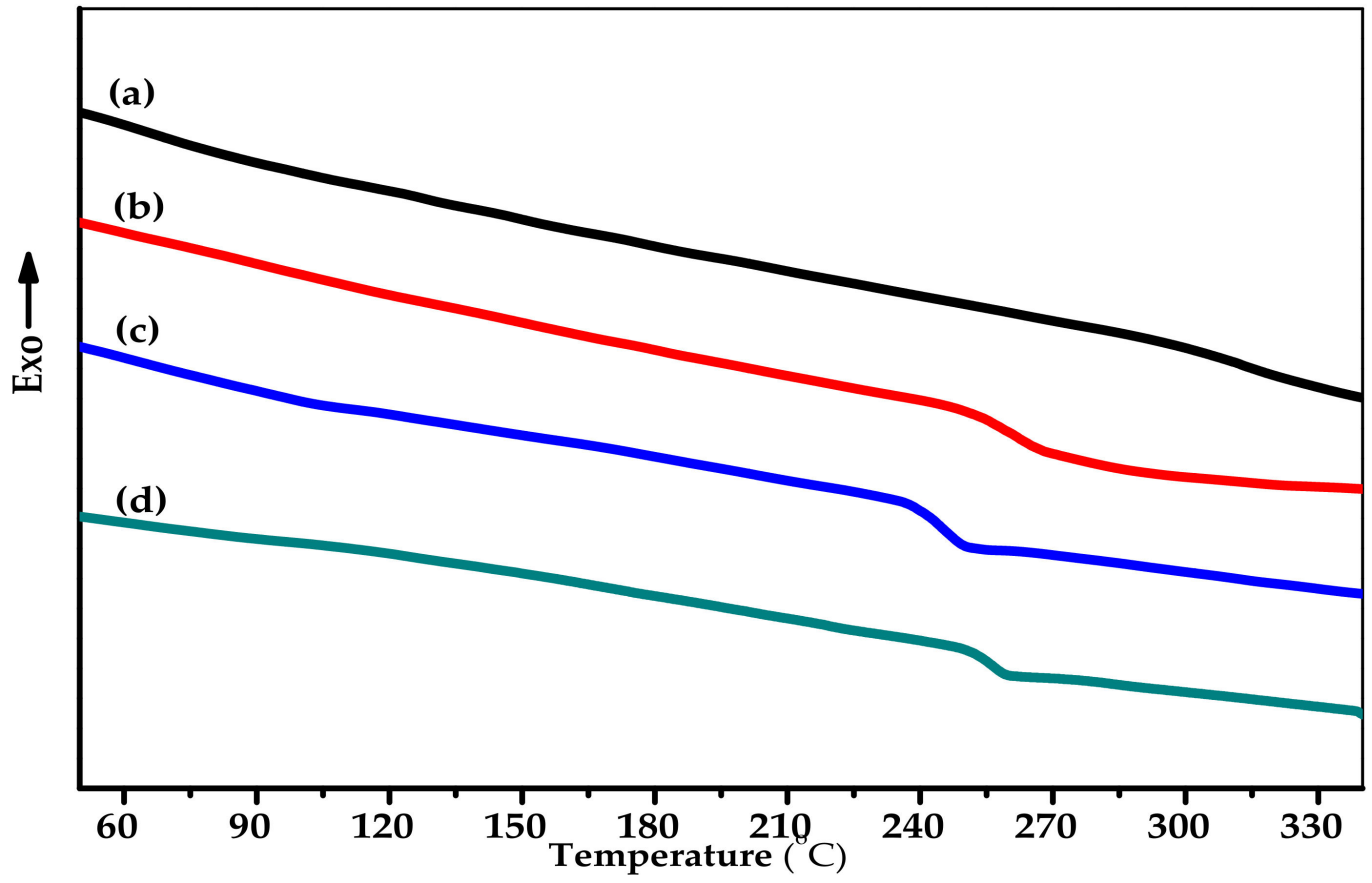
DSC thermograms of PIs: (**a**) CPI-PM, (**b**) CPI-BP, (**c**) CPI-6F, and (**d**) API-6F.

The thermogravimetric analyses (TGA) of CPI-PM, CPI-BP, CPI-6F, and API-6F are shown in Figure 4. These PIs exhibited degradation temperatures (*T*_d5_) higher than 450 °C (5% weight loss) with the char yields of 50%–65% at 800 °C (under nitrogen). The reference API-6F exhibited the highest *T*_d5_ indicating that the dimethyl moiety between phenylene groups was more stable than the cyclopentyl moiety between phenylene groups at high temperatures. Apart from that, polymer chain packing efficiency, and chain stiffness would also affect the *T*_g_ behavior. For cardo-type PIs with a hexafluoro dianhydride moiety, i.e., 6FDA and bulky pendent alicyclic groups on the polymer chains would cause various degrees of thermal transition changes. For example, PI main chains linked with bulky aliphatic hydrocarbon (diamantane) moieties [11], leading to a high *T*_g_ of 361 °C. Moreover, according to the contribution of different cardo groups, the *T_g_*’s of cardo-type PIs can be ranked in the following order [12]: diamantane (361 °C) > adamantane (296 °C) > norbornane (288 °C) > cyclododecane (276 °C). In this work, CPI-6F with a hexafluoro dianhydride moiety and cyclopentyl group on the polymer chain exhibited a *T*_g_ of 245 °C because of smaller size bulky group. Moreover, *T*_g_’s in the range of 245–313 °C were observed for the cardo-type PIs (Figure 5). CPI-PM with rigid phthalimide units exhibited the highest *T*_g_, whereas the lowest *T*_g_ was observed on CPI-6F with flexible hexafluoroisopropylidene linkages. For the reference sample, API-6F possessed a *T*_g_ of 255 °C that was slightly higher than that (245 °C) of CPI-6F. This implies that the five-membered ring cardo unit on the polymer backbone would bring about loose molecular packing. Thermal characteristics of these PIs are summarized in Table 2.

**Figure 6:**
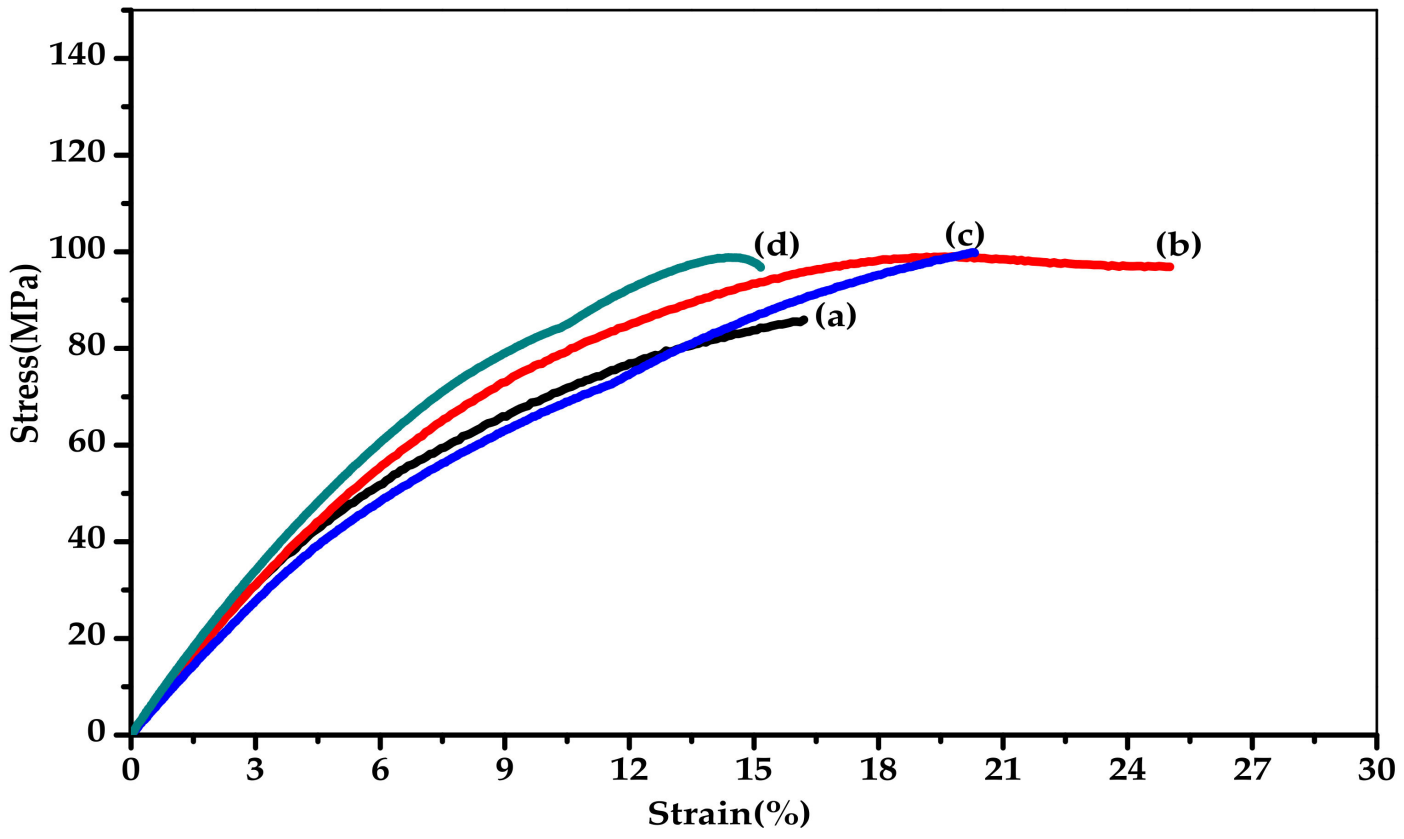
Tensile properties of the PIs: (**a**) CPI-PM, (**b**) CPI-BP, (**c**) CPI-6F, and (**d**) API-6F.

Tensile properties of these cardo-type PIs are shown in Figure 6. Tensile strengths are in the range of 85.0–99.4 MPa, whereas moduli are ranged from 913 to 1039 MPa. API-6F exhibited the highest Young’s modulus of 1039 MPa with a relatively small elongation of 14.2%, whereas CPI-6F exhibited a Young’s modulus of 913 MPa with an elongation of 20.0%. A longer elongation of 25.4% was also observed for CPI-BP. This implies that the incorporation of cyclopentyl cardo group into PI backbone will sacrifice a certain degree of dense packing but gain more toughness. The cardo-type PIs in this study exhibited reasonably good tensile strength and elongation at break. These tensile properties are comparable to those of PIs reported in literatures [11,12,13,41]. Moreover, all the cardo PIs exhibited large elongation at break in the range of 16%–27%. It is worth noting that the reported cardo-type PIs with larger bulky groups exhibited much smaller elongation in a range of 5.2%–7% [11,12]. With the reduction in size for cyclopentyl cardo groups, relatively flexible PIs could be achieved in this work. Furthermore, the tensile strength of API-6F was similar to that of CPI-6F. However, the elongation at break for API-6F was only 14.7%, which is much smaller than those of CPI-6F and CPI-BP. This indicates that five-membered rings of cardo between two phenylenes play an important role to enhance the toughness for polyimides.

### 3.2. Change of Average Interchain Distance (d-spacing)

**Figure 7:**
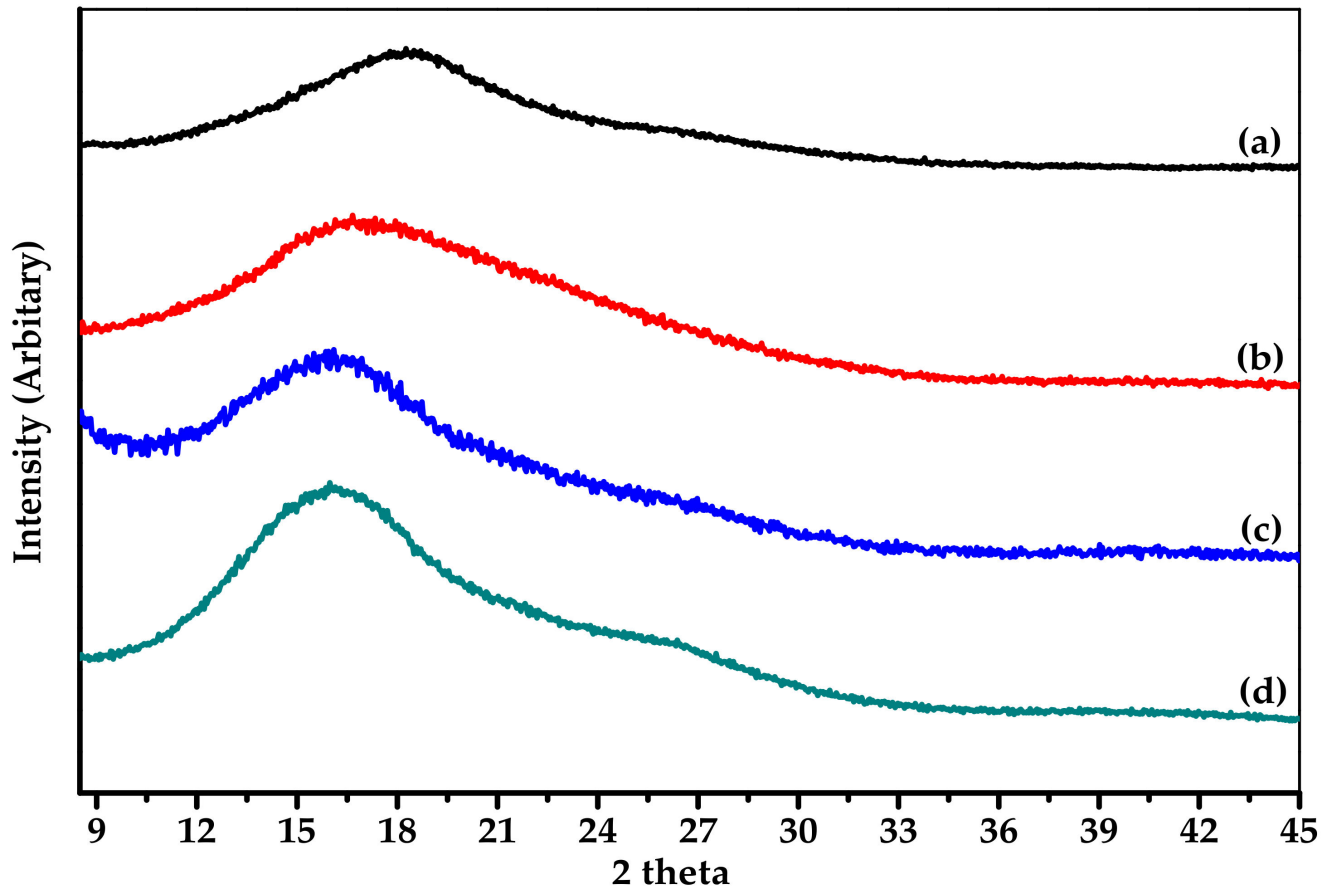
Wide-angle X-ray diffraction (WAXD) patterns of PIs: (**a**) CPI-PM, (**b**) CPI-BP, (**c**) CPI-6F, and (**d**) API-6F.

**Figure 8:**
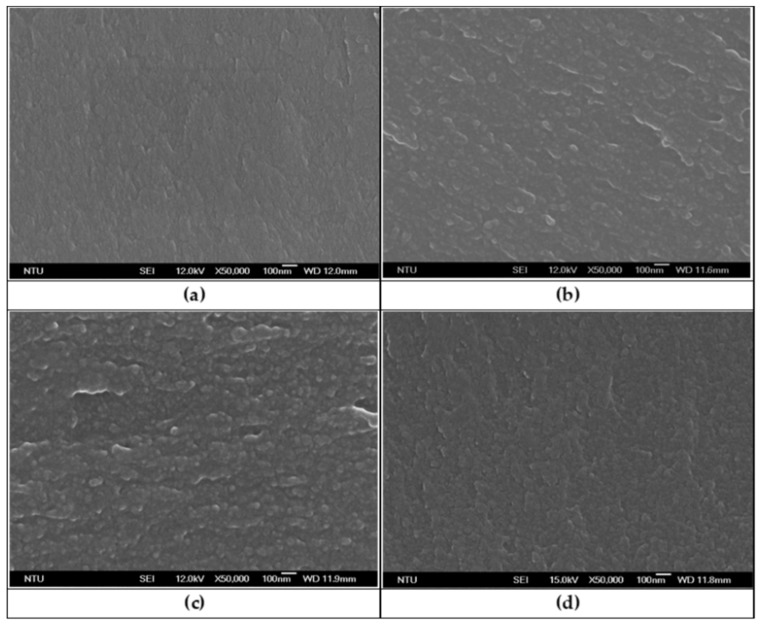
SEM micrographs of PI cross sections: (**a**) CPI-PM, (**b**) CPI-BP, (**c**) CPI-6F, and (**d**) API-6F.

Cardo type PIs were also investigated through wide-angle X-ray diffraction (WAXD; Figure 7). The diffraction patterns of all PIs exhibited broad shapes in the 2θ range of 10°–20° (band I) and 25°–30° (band II). This indicates these PIs are in amorphous state without crystalline phase [42]. These two broad peaks on each X-ray pattern were attributed to the intersegmental interference [43]. The value of average intersegmental distance, namely mean interchain distance (*d*-spacing) was calculated according to Bragg’s law: nλ = 2*d*sinθ. The broad diffraction 2θ was located at 16.10° (*d*-spacing = 5.51 Å) for CPI-6F (Figure 7c). The *d*-spacing = 5.51 Å was lower than that (5.76 Å; 16.10°) of the reported diamantane-based PI [11]. This is because the diamantane-cage would bring about a large void volume than would the cyclophentyl group within the polymer chains. In addition, the *d*-spacing of CPI-6F (5.51 Å) is larger than that of API-6F (5.46 Å). This implies that CPI-6F possessed rather loose chain packing feature and larger free volume when compared with API-6F in this study. A lower *d*-spacing for CPI-PM and CPI-BP is attributed to a relatively efficient chain-to-chain packing (Figure 7a,b). The relatively dense chain packing in CPI-PM and CPI-BP is consistent with the result of DSC study. The *d*-spacings of PIs are summarized in Table 2. In addition, the SEM micrographs from PI cross sections are shown in Figure 8. CPI-PM exhibited continuous and porous-free polymeric layer under 50,000× magnitude, whereas CPI-6F exhibited a porous-like morphology. The morphologies of these PIs were similar to those of dense PIs such as Matrimid [44], or crosslinked PI [41]. Consequently, specific voids were not easy to observe when compared with MOF based PIs [45,46] or PIM PIs [44,47].

### 3.3. Gas Permeation Properties of Cardo-Type Polyimides

Generally, gas transport through non-porous PI membranes can be regarded as the solution-diffusion process, in which permeability (P_A_) of a gas is the product of diffusion coefficient (D_A_) and solubility coefficient (S_A_):

P_A_ = D_A_ S_A_

**Figure 9:**
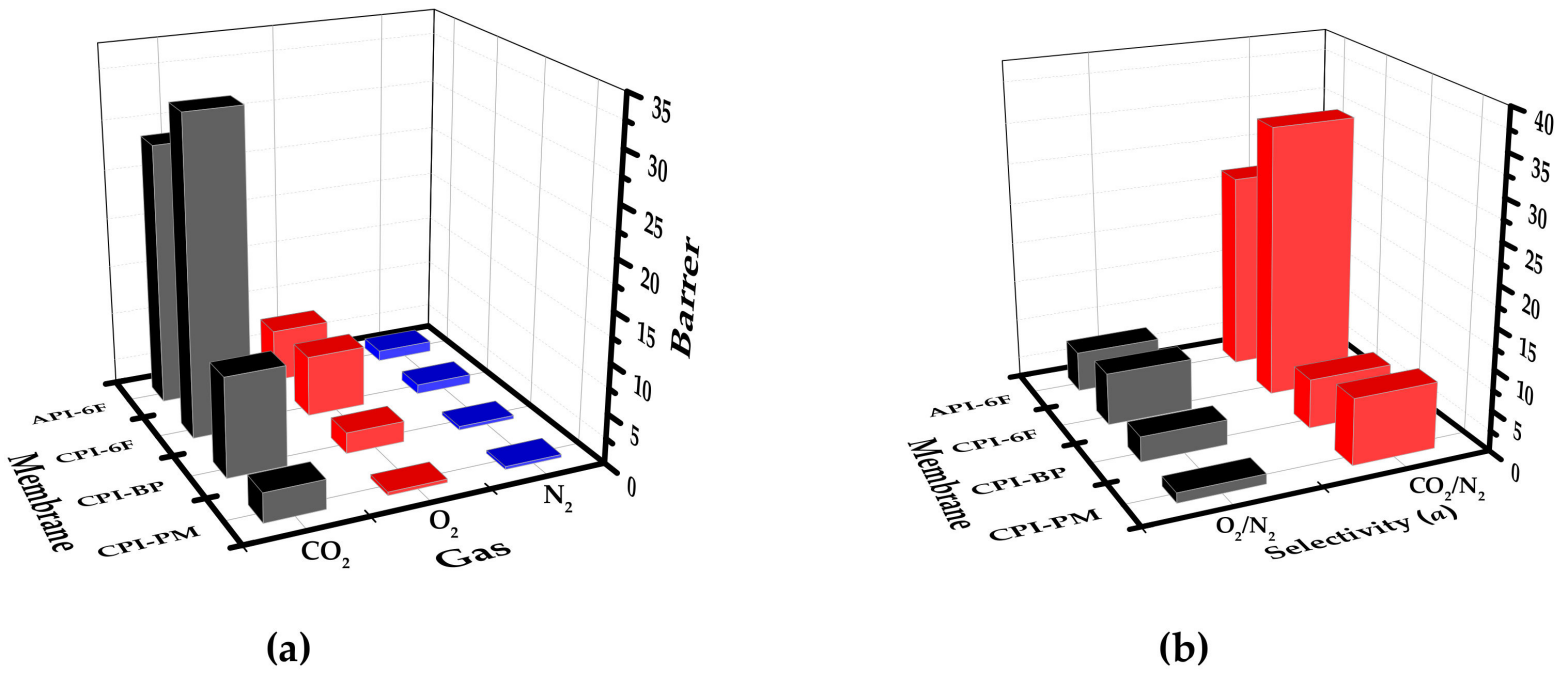
3D plot for comparison the performance of PI membranes: (**a**) Permeability: CO_2_ (black), O_2_ (red), N_2_ (blue), (**b**) selectivity (α): O_2_/N_2_ (black), CO_2_/N_2_ (red).

The selectivity (α_A/B_) of gas A to gas B is also the product of diffusivity (D) and solubility (S). For the pure-gas permeation analysis of the membranes for these PIs, the intermediate upstream pressure of 1 bar were chosen for O_2_, N_2_, CO_2_ studies. The permeability and selectivity results of gas pairs are shown in Table 3 and Figure 9. Based on the kinetic diameter of CO_2_ (3.3 Å), O_2_ (3.46 Å), N_2_ (3.64 Å) [48], and the *d*-spacing values of these PI membranes, these three gases were permeating through the PI membranes. Gas transport behavior for CPI-PM, CPI-BP, CPI-6F, and API-6F were investigated, respectively. The permeability of O_2_ for these three membranes exhibited the following order: CPI-6F (6.1 Barrer) > API-6F (5.33 Barrer) > CPI-BP (2.10 Barrer) > CPI-PM (0.36 Barrer). Moreover, the O_2_/N_2_ selectivity of CPI-6F was 5.2 times as much as that of CPI-PM. CPI-PM exhibited a lower O_2_ permeability because of its rather dense chain packing. The gas permeability behavior of CPI-PM is also consistent with the results of high *T*_g_ in DSC and smaller *d*-spacing value in XRD investigation. Similar behaviors were also observed for CO_2_ permeability and selectivity. CPI-6F possessed a higher CO_2_ permeability (31.77 Barrer) than API-6F (26.28 Barrer), whereas the selectivities of CO_2_/N_2_ were 33.44, 24.30 for CPI-6F, API-6F, respectively. Furthermore, D and S are also responsible for membrane performance. CO_2_ adsorption-desorption for these PIs was measured with a microbalance. The solubility of CO_2_ in CPI-6F was 7.28 cm^3^ (STP)/(cm^3^·cmHg), and the corresponding diffusivity of CO_2_ obtained from P = D × S was 4.36 × 10^−8^ cm^2^ s^−1^. Relatively large solubility and diffusivity were consistent with higher CO_2_ permeability for CPI-6F than API-6F with CO_2_ solubility of 5.49 cm^3^ cm^−3^ cmHg^−1^ and diffusivity of 4.78 × 10^−8^ cm^2^ s^−1^. Furthermore, the fractional free volume (FFV) of PIs was estimated by Bondi’s method [31,32,33,34]. CPI-6F exhibited not only the highest FFV of 18.9, but the highest gas permeability as well (Table 3). This also indicates that the presence of five-membered rings on polyimide backbones exerted a positive effect on gas membranes.

## 4. Conclusion and Outlook

The five-membered ring cardo PIs, such as CPI-PM, CPI-BP, and CPI-6F, were successfully developed based on a diamine (CPDA) derived from a petroleum C5 byproduct DCPD. This cardo-type CPI-6F based on CPDA and 6FDA units possessed excellent processability (soluble in NMP, DMAc, THF and chloroform) and mechanical properties (tensile strength 99.4 MPa; 20.0% elongation). These cardo PIs were also evaluated as membranes for gas separation, and the CPI-6F with cardo substitution group exhibited superior O_2_ permeability (6.10 Barrer) and O_2_/N_2_ selectivity (6.44). It is because of the relative higher gas solubility and diffusivity for CPI-6F. This work demonstrates that robust polyimides can be achieved via a facile synthetic route utilizing DCPD, an abundant and inexpensive raw material.

## Figures and Tables

**Table 1:** Solubility and inherent viscosity of PIs.

Sample	Solubility *					Inherent Viscosity η_inh _(dL/g)
	NMP	DMF	DMAc	THF	CHCl_3_	
**CPI-PM**	--	--	--	--	--	0.99
**CPI-BP**	+h	--	--	--	--	1.27
**CPI-6F**	+h	+h	Δ	Δ	+h	0.85
**API-6F**	+h	+h	Δ	Δ	--	0.90

* Qualitative solubility measured with 100 mg of the polymer in 2 mL of solvent. +h, soluble at elevated temperatures; Δ, partially soluble at elevate temperature; --, insoluble.

**Table 2:** Thermal and mechanical properties of cardo-type PIs.

Sample	Thermal Properties ^a,b^		Mechanical Properties ^c^			WAXD Pattern ^d^	
	*T*_g_ (°C)	*T*_d5_ (°C)	Tensile Strength at Break (MPa)	Elongation at Break (%)	Modulus (MPa)	2θ (°)	*d*-spacing (Å)
CPI-PM	313	467	85.0 ± 0.4	15.7 ± 0.5	972 ± 50	18.50	4.80
CPI-BP	262	461	96.7 ± 1.1	25.4 ± 1.5	949 ± 10	16.66	5.32
CPI-6F	245	464	99.4 ± 0.2	20.0 ± 0.3	913 ± 15	16.10	5.51
API-6F	255	474	95.8 ± 1.7	14.2 ± 1.0	1039 ± 45	16.15	5.46

^a^ Glass transition temperature (*T*_g_) was measured by DSC at a heating rate of 10 °C/min. ^b^ Decomposition temperature (*T*_d5_) at 5 wt % of weight loss. ^c^ Averages were taken from three measurements. ^d^ The *d-*spacing value is typical for the average chain-chain distance calculated from the prominent peak in WAXD pattern by Bragg equation (nλ = 2*d*sinθ).

**Table 3:** Gas permeability and selectivity of PI membranes at 35 °C and 1 bar upstream pressure.

Sample	Permeability (Barrer)			Selectivity (α_A/B_)		*V*_f_^a^	FFV (%) ^b^
	CO_2_	O_2_	N_2_	O_2_/N_2_	CO_2_/N_2_		
CPI-PM	2.94	0.36	0.29	1.24	8.16	63.6	12.7
CPI-BP	10.00	2.10	0.32	3.09	6.09	89.5	15.3
CPI-6F	31.77	6.10	0.95	6.44	33.44	126.7	18.9
API-6F	26.28	5.33	1.07	4.98	24.30	116.5	18.1

^a,b^ Free volume (*V*_f_) and fractional free volume (FFV) were obtained from ref [31,32,33,34].

## Supplementary Materials

The following are available online at https://www.mdpi.com/2073-4360/11/12/2029/s1.
