# Supplementary material for: Synthesis and Properties of Cyclopentyl Cardo-Type Polyimides Based on Dicyclopentadiene
Source: Polymers (Basel). 2019 Dec 6;11(12):2029. doi: 10.3390/polym11122029 (PMC6960653; doi:10.3390/polym11122029)
Supplement: Supplementary file 1 [file polymers-11-02029-s001.pdf]

Supporting Information

Synthesis and Properties of Cyclopentyl Cardo-Type Polyimides Based on Dicyclopentadiene

Shih-Chieh Yeh[1,2], Jen-Yu Lee[1], Chung-Ta Hsieh[1], Ya-Chin Huang[1], Kuan-Syun Wang[1], Chien-Hsin Wu[1,2], Chien-Chieh Hu[3*], Shu-Chen Chiang[4], Ru-Jong Jeng[1,2*]*

1. Institute of Polymer Science and Engineering, National Taiwan University, Taipei 106, Taiwan
2. Graduate Institute of Applied Science and Technology, National Taiwan University of Science and Technology, Taipei 106, Taiwan
3. Graduate Institute of Applied Science and Technology, National Taiwan University of Science and Technology, Taipei 106, Taiwan
4. Chung Shan Institute of science and technology, Taoyuan 325, Taiwan

Fig. S1. ^{1}H-NMR spectrum of BPAA (DMSO-d_6).

Fig. S2. ^{13}C-NMR spectrum of BPAA ($CDCl_3$).

Fig. S3. ^{1}H-NMR spectrum of CPDA (DMSO-d_6).

Fig. S4. ^{13}C-NMR spectrum of CPDA ($CDCl_3$)

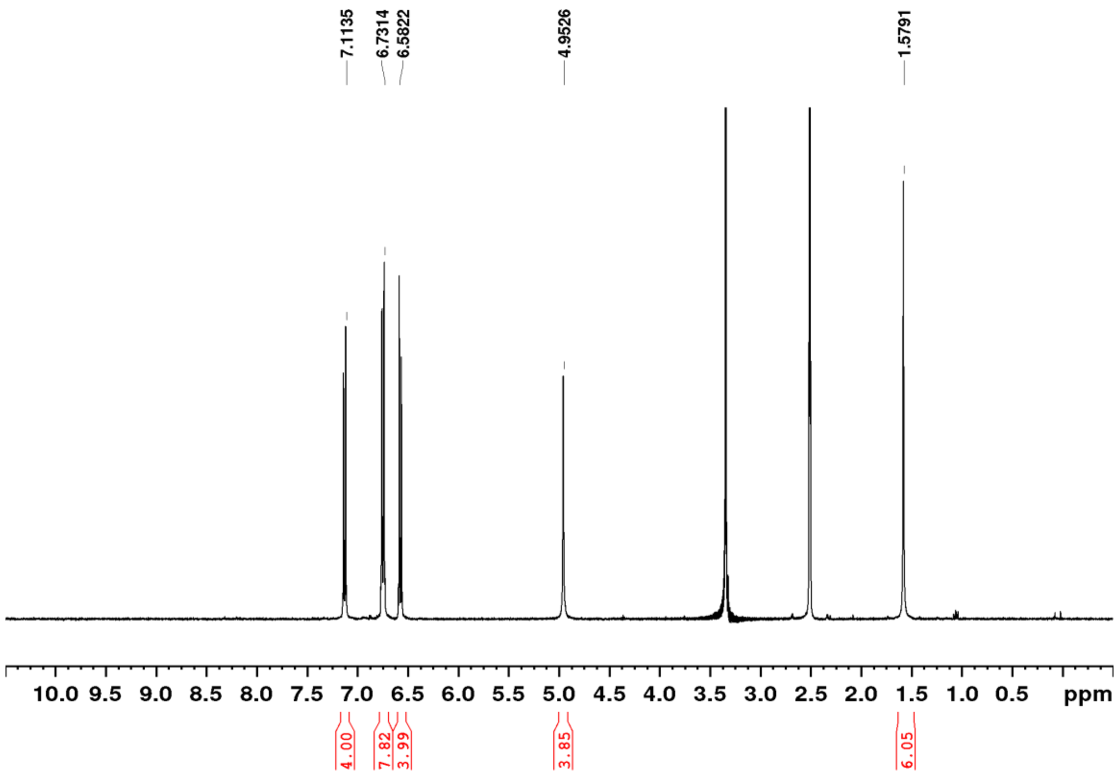

Fig. S1. ^{1}H-NMR spectrum of BPAA (DMSO-*d6*).

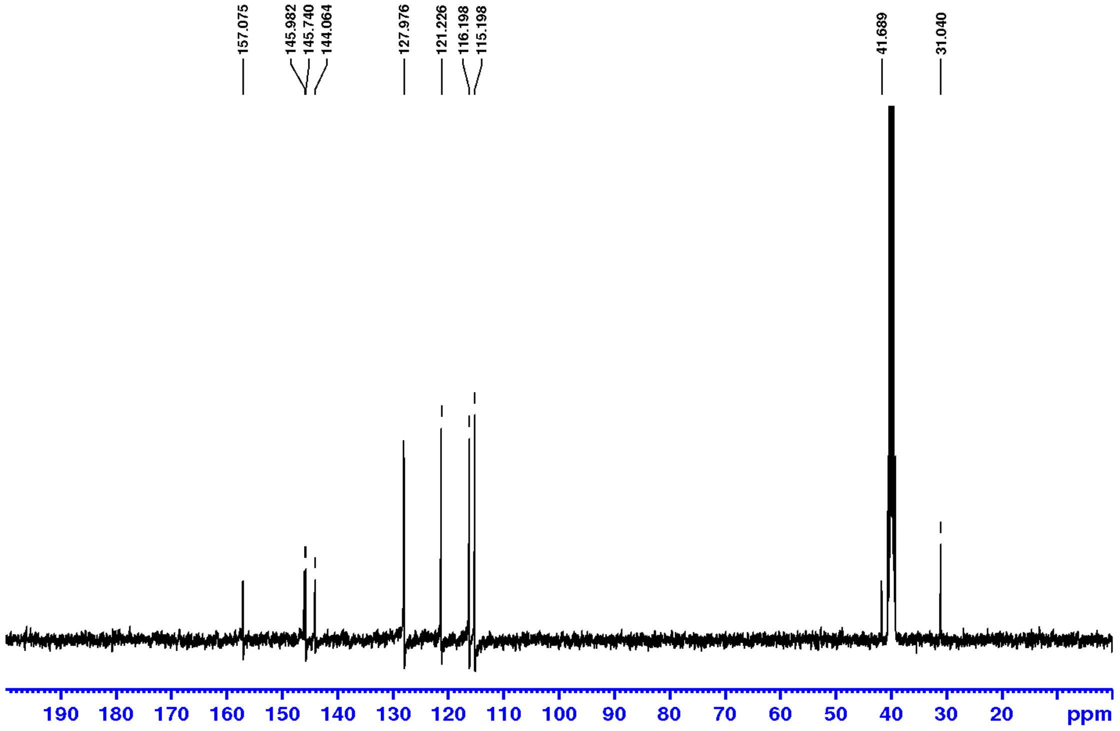

Fig. S2. ^{13}C-NMR spectrum of BPAA ($CDCl_3$).

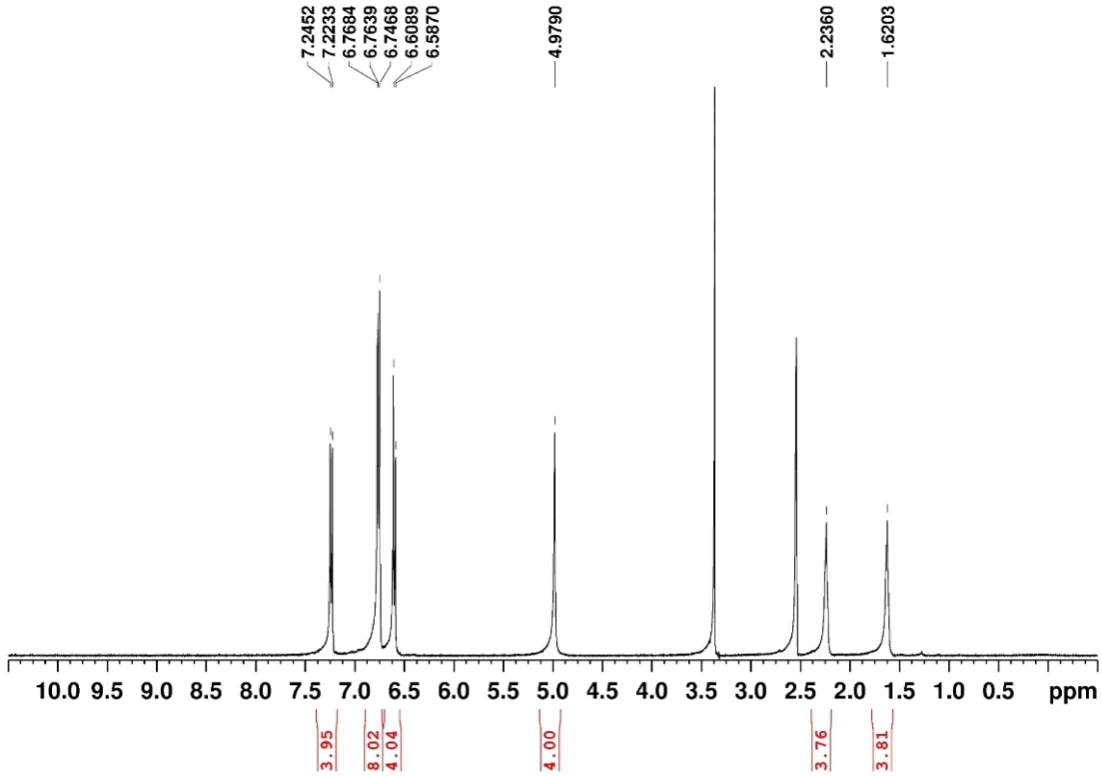

Fig. S3. ^{1}H-NMR spectrum of CPDA (DMSO-*d6*).

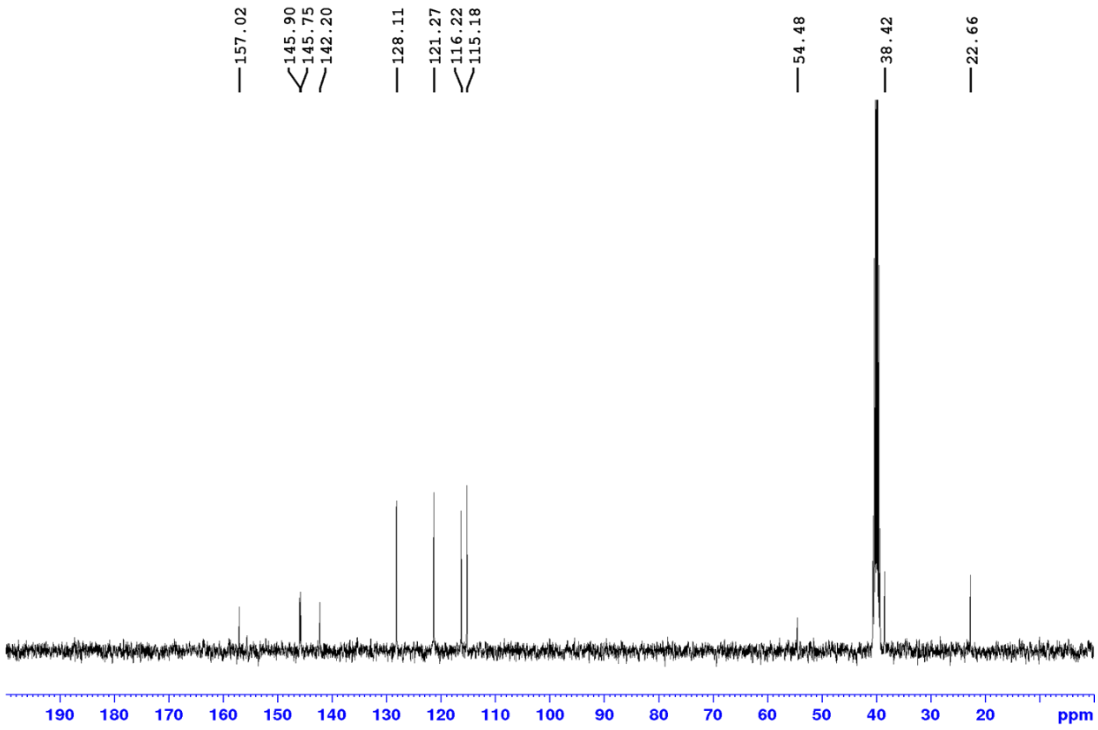

Fig. S4. ^{13}C-NMR spectrum of CPDA ($CDCl_3$).
